# Integrated transcriptomic analysis of distance-related field cancerization in rectal cancer patients

**DOI:** 10.18632/oncotarget.17864

**Published:** 2017-05-15

**Authors:** Honglin Guo, Weigen Zeng, Lin Feng, Xuexin Yu, Ping Li, Kaitai Zhang, Zhixiang Zhou, Shujun Cheng

**Affiliations:** ^1^ State Key Laboratory of Molecular Oncology, Department of Etiology and Carcinogenesis, National Cancer Center/Cancer Hospital, Chinese Academy of Medical Sciences and Peking Union Medical College, Beijing 100021, China; ^2^ Department of General Surgery, Beijing Chao-Yang Hospital, Capital Medical University, Beijing 100020, China; ^3^ College of Bioinformatics Science and Technology, Harbin Medical University, Harbin 150081, China; ^4^ Department of Colorectal Surgery, National Cancer Center/Cancer Hospital, Chinese Academy of Medical Sciences and Peking Union Medical College, Beijing 100021, China

**Keywords:** field cancerization, CRC, fatty acid metabolism, ribosome biogenesis, CPT2

## Abstract

Field cancerization (FC) occurs in various epithelial carcinomas, including colorectal cancer, which indicates that the molecular events in carcinogenesis might occur in normal tissues extending from tumors. However, the transcriptomic characteristics of FC in colorectal cancer (CRC) remain largely unexplored. To investigate the changes in gene expression associated with proximity to the tumor, we analyzed the global gene expression profiles of cancer tissues and histologically normal tissues taken at various distances from the tumor (1 cm, 5 cm and the proximal end of the resected sample) from 32 rectal cancer patients. Significantly differentially expressed genes related to the distance from the tumor were screened by linear mixed effects analysis using the lme4 package in R. The distance-related differentially expressed genes that were gradually up-regulated (n=302) or gradually down-regulated (n=568) from normal tissues to the tumor were used to construct protein-protein interaction (PPI) networks. Three subnetworks among the gradually up-regulated genes and four subnetworks among the gradually down-regulated genes were identified using the MCODE plugin in the Cytoscape software program. The most significantly enriched Gene Ontology (GO) biological process terms were “ribosome biogenesis”, “mRNA splicing via spliceosome”, and “positive regulation of leukocyte migration” for the gradually up-regulated subnetworks and “cellular calcium ion homeostasis”, “cell separation after cytokinesis”, “cell junction assembly”, and “fatty acid metabolic process” for the gradually down-regulated subnetworks. Combined with the previously constructed multistep carcinogenesis model used for the analysis, 50.59% of the genes in the subnetworks (43/85) displayed identical changes in expression from normal colon tissues to adenoma and colon cancer. We focused on the 7 genes associated with fatty acid metabolic processes in the distance-related down-regulated subnetwork. Survival analysis of patients in the CRC dataset from The Cancer Genome Atlas (TCGA) revealed that higher expression of these 7 genes, especially CPT2, ACAA2 and ACADM, was associated with better prognosis (*p* = 0.034, *p* = 0.00058, *p* = 0.039, *p* = 0.04). Cox proportional hazards regression analysis revealed that CPT2 was an independent prognostic factor (*p* = 0.004131). Our results demonstrate that field cancerization occurs in CRC and affects gene expression in normal tissues extending from the tumor, which may provide new insights into CRC oncogenesis and patient progression.

## INTRODUCTION

Colorectal cancer (CRC) is the third most common cancer in both men and women [1]. It is commonly accepted that CRC develops through a multistep carcinogenesis process from normal colorectal epithelium to adenoma, which then progresses to cancer, accompanied by the accumulation of molecular alterations [2]. The molecular changes that give rise to the cancer can occur long before the morphological abnormality of the tissue. Studies of such early molecular alterations could provide valuable information in risk assessment, early cancer detection and monitoring of progression in cancer management.

In carcinogenesis, the concept of field cancerization (FC, also known as field carcinogenesis, field effect, field defect) was proposed by Slaughter et al. in 1953 in a study of normal tissues in oral squamous cell carcinoma [3]. FC refers to the phenomenon in which histologically adjacent normal tissues have molecular alterations similar to those of the tumor itself. That is, the aberrant molecular alterations and environmental modifications are present throughout the organ that gives rise to the tumor [4]. This phenomenon has been studied in several epithelial tumors, including non-small cell lung cancer [5, 6], colorectal cancer [7, 8], breast cancer [9], head and neck cancer [10] and prostate cancer [11, 12].

The colorectum has a continuous epithelium that is exposed to environmental substances, including carcinogens, and thus provides an ideal model to study FC. A gradient of carcinoembryonic antigen (CEA) expression has been observed in normal tissues adjacent to CRC [13]. Recent studies of FC have revealed that genetic and epigenetic changes are common in normal tissues adjacent to the tumor in CRC. Normal tissues surrounding the tumor to distances as far as 10 cm exhibit chromosomal instability [14]. Nuclear abnormalities present in normal-appearing tissue in the field of CRC include chromatin compaction and rearrangement [8]. Galandiuk et al. observed the same mutation in TP53 in both cancer and normal epithelium [15]. Aberrant DNA methylation has been identified as a potential biomarker of FC in the colon. Hypermethylation of the O^6^-methylguanine-DNA methyltransferase gene (MGMT) promoter has been observed in normal tissues from CRC patients [16–18] and favors transition mutations in P53 and KRAS, suggesting an association with the progression of CRC. Other reported hypermethylated genes observed in normal tissues of CRC patients include SFRP2, TFPI2, NDRG4, BMP3 [7] and ADAMTS14 [16]. In addition to genetic and epigenetic changes, Facista et al. observed expression deficiencies in the DNA repair proteins Pms2, Ercc1 and Xpf in approximately 1 million crypts near cancers, providing evidence of FC at the protein level [19]. Notably, promoter methylation of MGMT in CRC is also associated with the adenoma-carcinoma sequence [20], indicating it might act as an early event in the oncogenesis of CRC.

Although considerable progress has made in profiling the genetic and epigenetic changes in FC of CRC, the transcriptome of histologically normal tissues in FC has not been clearly characterized. In this study, we performed integrated bioinformatics analysis of global gene expression profiles of matched cancer tissues and adjacent histologically normal tissues taken at various distances from the tumor (1 cm, 5 cm and the proximal end of the resected sample) to define the transcriptomic characteristics of distance-related FC in rectal cancer patients. Combined with a multistep carcinogenesis model, we report the key function changes in FC and identify carnitine palmitoyltransferase II (CPT2) as an independent prognostic factor in CRC patients, offering new insights into oncogenesis and patient progression.

## RESULTS

### Global gene expression profile analysis of field cancerization in rectal cancer patients

**Figure 1:**
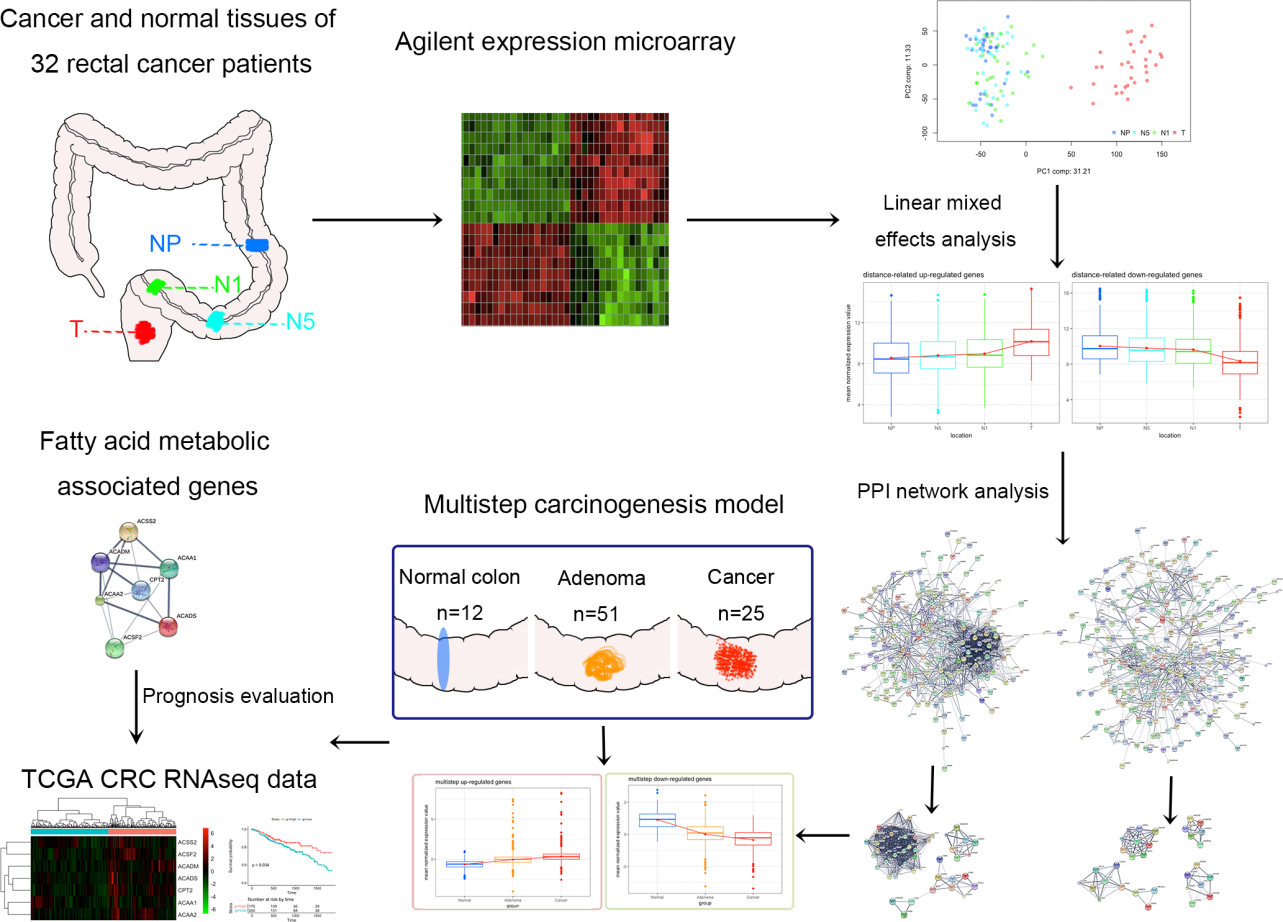
Schematic representation of the stepwise selection and evaluation of genes with distance-related expression patterns Cancer tissues and corresponding adjacent histologically normal tissues were obtained from 32 rectal cancer patients who underwent surgery. The samples obtained from each patient included primary rectal cancer tissue (T) and adjacent histologically normal tissues taken 1 cm from the tumor (N1), 5 cm from the tumor (N5) and as far as possible from the tumor (proximal end of the resected sample, NP).

To illustrate distance-related FC, we collected tumor tissues and paired normal tissues from specimens surgically resected from 32 rectal cancer patients (one patient had colon cancer in addition to rectal cancer). The paired adjacent histologically normal tissues were collected at three different distances from the tumors as follows: 1 cm from the tumor (N1), 5 cm from the tumor (N5) and as far as possible from the tumor (proximal end of the resected sample, NP). The gene expression profiles of all 128 samples were analyzed. The clinical characteristics of the patients are summarized in Table 1. Detailed clinical information for the patients, including the exact distance of NP from the tumor, is presented in Supplementary Table 1. A schematic representation of the study design is shown in Figure 1.

**Table 1:** Clinical characteristics of the patients

Clinical characteristic	No. of patients (%)
Total no.	32 (100)
Age (year)	
≥ 60	17 (53)
< 60	15 (47)
Gender	
Male	19 (59)
Female	13 (41)
TNM stage	
I + II	15 (47)
III + IV	17 (53)
Differentiation	
Well and mod	28 (88)
Poorly	40 (12)
Polyps	
With	14 (44)
Without	18 (56)

After data preprocessing, we performed a principle component analysis (PCA) and hierarchical clustering of the data to observe the overall differences in expression in the samples (Supplementary Figure 1). The difference between cancer and normal tissues is more obvious than individual differences, as all cancer samples are clustered together and are separate from the normal samples. Then, we conducted a linear mixed effects analysis to identify the distance-related differentially expressed genes using a false discovery rate (FDR) cutoff < 0.0001, which yielded 870 genes, of which 302 were gradually up-regulated (Supplementary Table 2) and 568 were gradually down-regulated (Supplementary Table 3) from normal tissues to tumors (Supplementary Figure 2). We considered these distance-related differentially expressed genes as representing FC in rectal cancer patients for further analysis.

### Protein-protein interaction network analysis of the distance-related differentially expressed genes

**Figure 2:**
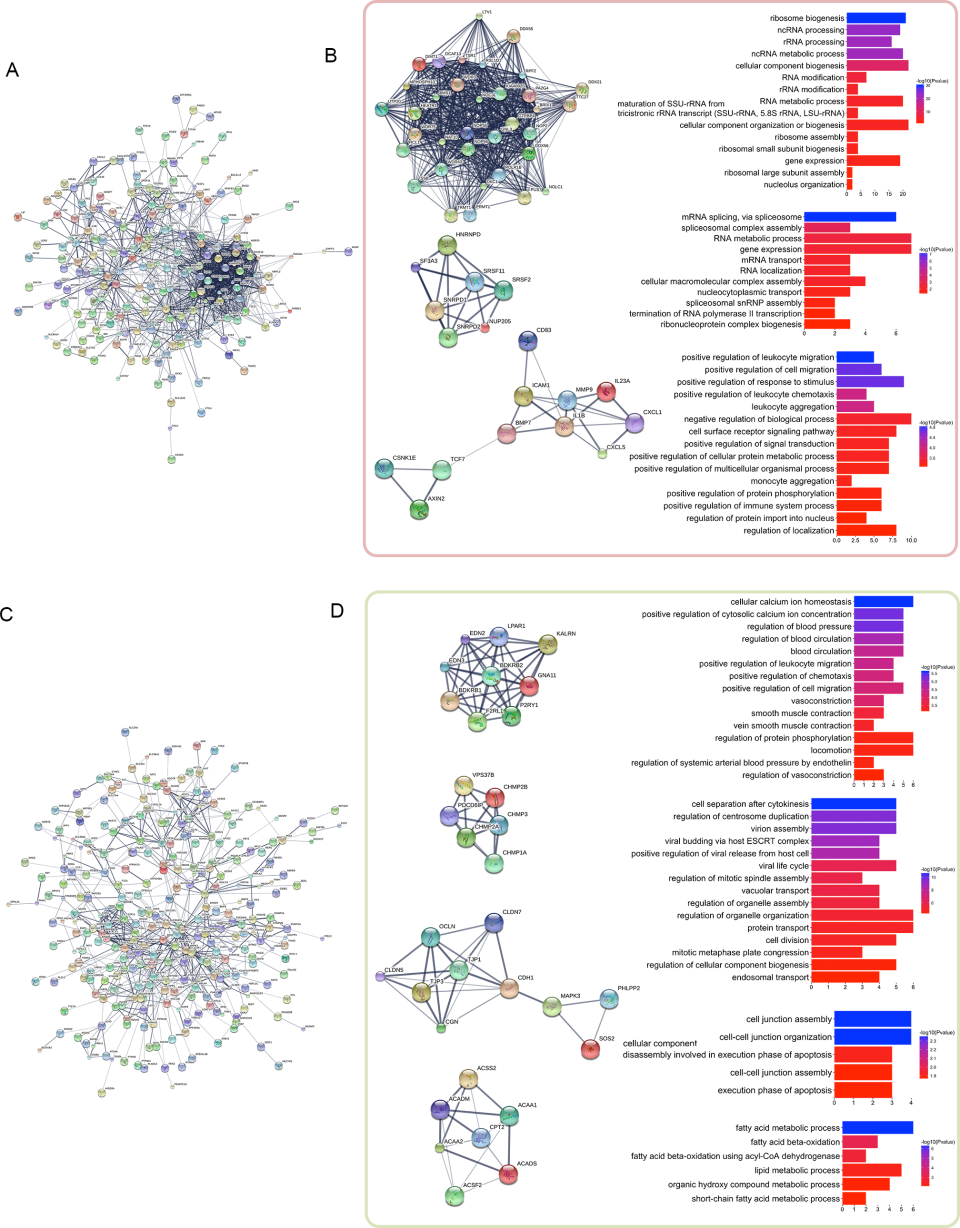
PPI network analysis of the distance-related differentially expressed genes The distance-related differentially expressed genes were imported into STRING to construct PPI networks. The largest connected subnetworks of distance-related up-regulated genes **(A)** and down-regulated genes **(C)** are shown. The left columns of **(B)** and **(D)** show the discrete clusters identified from the network, whereas the corresponding right columns show the most significant enriched GO terms for each cluster.

**Figure 3:**
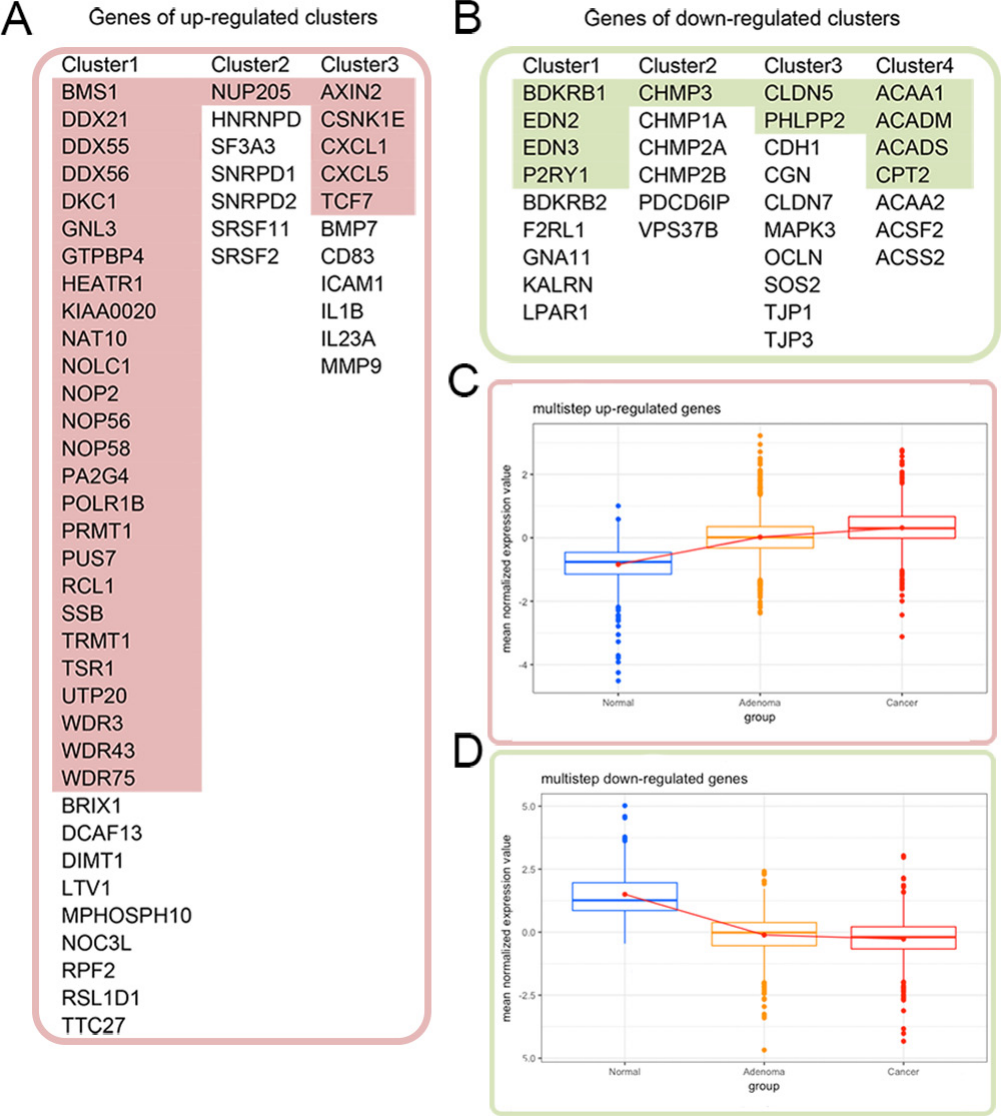
Cross validation of the gene expression patterns in the multistep carcinogenesis model **(A)** and **(B)** list the genes of the distance-related up-regulated and down-regulated clusters, respectively. The colored genes in **(A)** and **(B)** exhibited identical up- and down-regulation trends from normal colon mucosa through adenoma to colon cancer. Their expression patterns are shown in **(C)** and **(D)**, respectively, with red color for up-regulation in the tumor and green color for down-regulation.

**Figure 4:**
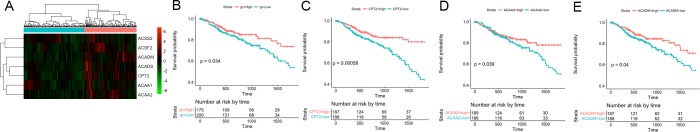
Five-year survival analysis of the 7 fatty acid metabolism-associated genes in the TCGA CRC dataset **(A)** Hierarchical clustering of the 7 fatty acid metabolism-associated genes from the TCGA CRC dataset. The expression levels of the genes are illustrated as a color spectrum, with red, black and green representing high, medium and low expression, respectively, in a matrix indexed by genes in rows and samples in columns. The samples were divided into two groups. **(B)** Kaplan-Meier survival analysis and log-rank test of these 7 genes, using hierarchical clustering of their expression values as the categorical variable to divide the 375 patients into two groups. **(C)**, **(D)** and **(E)** show the Kaplan-Meier survival curves for CPT2, ACAA2 and ACADM, respectively.

We used these distance-related up-regulated genes (302) and down-regulated genes (568) to construct PPI networks in STRING. The largest connected subnetwork for the up- and down-regulated PPI networks contained 208 nodes (Figure 2A) and 287 nodes (Figure 2C), respectively. The node degree of both networks follows a power law distribution (Supplementary Figure 3). We then extracted discrete clusters from the subnetworks using the MCODE plugin in Cytoscape software. There were 3 clusters in the up-regulated subnetwork (left column of Figure 2C) and 4 clusters in the down-regulated subnetwork (left column of Figure 2D), which included a total of 85 genes. The gene members of the 3 up-regulated clusters and 4 down-regulated clusters are listed in Figure 3A and 3B, respectively. These 7 clusters were considered the most relevant gene sets of FC related to tumor proximity.

We then examined the functions of these 7 clusters using Gene Ontology (GO) enrichment analysis. Up to 15 of the most significantly enriched GO biological process terms corresponding to the clusters are shown in the right columns of Figure 2C and 2D. The enriched functions of these clusters are centralized. The most significantly enriched terms were “ribosome biogenesis”, “mRNA splicing via spliceosome”, and “positive regulation of leukocyte migration” for the gradually up-regulated cluster 1 to cluster 3, respectively, and “cellular calcium ion homeostasis”, “cell separation after cytokinesis”, “cell junction assembly”, and “fatty acid metabolic process” for the gradually down-regulated cluster 1 to cluster 4, respectively. These functions are likely closely related to FC with proximity to the tumor in rectal cancer patients.

### Cross validation of expression pattern of distance-related differentially expressed genes in the multistep carcinogenesis model

In addition to the distance-related FC model we built in this study, our laboratory previously constructed a multistep carcinogenesis model composed of the gene expression profiles of 12 normal colon mucosa samples, 51 adenoma biopsy samples and 25 colon adenocarcinoma samples. Clinical characteristics of these samples are summarized in Supplementary Table 4. We then cross validated the distance-related differentially expressed genes in the multistep carcinogenesis model to investigate whether their expression trends from normal tissues to adenoma and cancer were similar. Among the 870 distance-related differentially expressed genes, 343 genes (39.43%) exhibited identical trends in up-regulated (131/302, 43.05%) or down-regulated (212/568, 37.32%) expression from normal tissues to cancer tissues in the two models. Among the 85 genes in the 7 clusters identified in the PPI networks, 43 genes (50.59%) had similar trends in expression from normal tissues to cancer tissues in the two models, of which 32 genes (red color in Figure 3A) were up-regulated and 11 genes (green color in Figure 3B) were down-regulated in cancer tissues. The expression patterns of these 43 genes in the multistep carcinogenesis model are shown in Figure 3C and 3D. Notably, of the 35 genes in the “ribosome biogenesis” GO term of the distance-related up-regulated cluster 1, 26 genes (74.29%) were also up-regulated in the tumor tissue in the multistep carcinogenesis model. This agreement indicates the importance of up-regulation of ribosome biogenesis in carcinogenesis. We considered the genes that were differentially expressed in both the distance-related FC model and multistep carcinogenesis model more relevant to cancer initiation and progression.

### Prognostic value of fatty acid metabolic process-associated genes

Given the impact of mitochondrion genome mutations on FC, we focused on fatty acid metabolic process-associated genes, which reflect the function of the mitochondrion, and evaluated their prognostic value in the CRC dataset from The Cancer Genome Atlas (TCGA) (Figure 4). Using hierarchical clustering of the 7 genes to divide the patients into two groups (Figure 4A), the 7-gene signature was found to be significantly associated with overall 5-year survival in 375 CRC patients (log-rank *p* = 0.034, Figure 4B). For each member of the 7-gene signature, we divided the samples into a higher expression group and a lower expression group based on the median gene expression value. Both the log-rank test and the univariate Cox regression analysis confirmed that lower expression of CPT2, ACAA2 and ACADM in cancer tissues could predict poor prognosis of CRC patients (Figure 4C, 4D and 4E). Multivariate Cox proportional hazards regression analysis validated CPT2 as an independent prognostic factor (*p* = 0.004131). The Cox proportional hazards regression analysis of the clinical characteristics (including age, gender and TNM stage), gene signature and each single gene of the TCGA CRC dataset is shown in Table 2.

**Table 2:** Cox proportional hazards regression analysis of overall survival in TCGA dataset, including gender, age, TNM stage and gene expression (n=375)

Factors		Univariate cox regression				Multivariate cox regression			
		HR	95% CI	*P*		HR	95% CI	*P*	
Gender	M/F	1.0871	0.6902-1.712	0.719		0.9357	0.5726-1.529	0.790835	
Age		1.028	1.009-1.048	0.0038	*	1.0385	1.0171-1.0604	0.000373	*
Stage	II/I	1.084	0.3928-2.99	0.876652		0.966	0.344-2.7107	0.947	
	III/I	3.007	1.1636-7.772	0.023043	*	2.789	1.0258-7.5823	0.044444	*
	IV/I	5.644	2.1162-15.051	0.000545	*	6.026	2.1317-17.0328	0.000705	*
gr^§^	Low/High	1.6626	1.035-2.671	0.0357	*	0.6903	0.3116-1.5294	0.361205	
CPT2		0.9985	0.9977-0.9993	0.000251	*	0.9984	0.9973-0.9995	0.004131	*
ACADM		0.9989	0.9982-0.9996	0.00318	*	0.9999	0.999-1.0007	0.775074	
ACAA2		0.9997	0.9995-0.9999	0.00577	*	0.9998	0.9995-1	0.068882	
ACAA1		1	0.9994-1	0.884		1	0.9995-1.0008	0.654016	
ACADS		0.9996	0.9992-1	0.149		1	0.9999-1.0011	0.127275	
ACSS2		0.9999	0.9997-1	0.469		0.9998	0.9996-1	0.111654	
ACSF2		1	0.9993-1	0.928		1	0.9992-1.0009	0.975314	

§Samples were divided into two groups based on the hierarchical clustering of gene expression of the 7 fatty acid metabolic associated genes (gr). *p<0.05. HR: hazard ratio; CI: confidence interval.

## DISCUSSION

In this study, we built a distance-related model to study the characteristics of the FC transcriptome in rectal cancer patients. We identified significantly differentially expressed genes with gradient distance-related expression trends from normal adjacent tissues to the tumor by linear mixed effects analysis. Moreover, we revealed the key function changes in FC by PPI network analysis, and half of these key function-related genes exhibited similar expression trends from normal colon mucosa to adenoma and cancer, supporting possible roles in CRC pathogenesis. In addition, higher expression of distance-related down-regulated fatty acid metabolism-associated genes was associated with the prognosis of CRC patients. CPT2 was shown to be an independent prognostic factor in predicting the overall survival of CRC patients.

CRC is an ideal model for investigating FC due to its continuous epithelium. Normal-appearing tissues adjacent to the tumor have epigenetic, proteomic and structural alterations in CRC patients [7, 8, 16, 21]. Using a similar study design, Hawthorn et al. compared transcript expression in a series of tumors and sites ranging from 1 to 10 cm distal to the tumor in 12 colon cancer patients [14]. Because they found no differentially expressed genes among the normal epithelial cell groups, we incorporated the tumor tissue as the 0 point in the distance-related model and identified 870 distance-related differentially expressed genes by linear mixed effects analysis. Multistep carcinogenesis models are used to study the molecular alterations at various stages of cancer development to identify early events during carcinogenesis. Such models are particularly important in CRC because it is commonly accepted that CRC develops through a normal-to-adenoma-to-cancer progression sequence [2]. Hypermethylation of the DNA repair gene MGMT is a widely studied FC marker that has been found in both precancerous lesions and normal-appearing cells adjacent to tumors [17, 20]. In our study, 39.43% (343/870) of the distance-related differentially expressed genes exhibited similar up- or down-regulation trends in tumors compared to the normal mucosa and adenomas. This result indicates that the gene expression changes in FC might be a useful resource for discovering potential uncharacterized molecules in CRC pathogenesis.

Our analyses of the key PPI clusters of the distance-related differentially expressed genes indicated significant up-regulation of ribosome biogenesis in the tumor, with 74.29% (26/35) of the members of the cluster exhibiting similar expression trends in both models (first column of Figure 3A). This high degree of consistency indicates the relationship between up-regulated ribosome biogenesis and cancer onset. Ribosomes are organelles that function in protein synthesis, which is one important step in the central dogma of molecular biology, i.e., translation. The ribosome biogenesis process includes ribosome DNA transcription in the nucleus, ribosome RNA (rRNA) assembly in the nucleoplasm and, finally, ribosome completion in the cytoplasm [22]. In addition to its important role in cellular physiological processes, growing evidence suggests that up-regulated ribosome biogenesis has a close relationship with cancer [23]. Based on the necessity of efficient ribosome biogenesis for the increasing demand of protein synthesis in the cell cycle, ribosome biogenesis regulates cell cycle progression in proliferating cells [24, 25]. The stimulatory factors that regulate the cell cycle also affect ribosome production [26]. Recent studies have found that the rate of ribosome biogenesis controls the expression and activity of the tumor suppressor p53; that is, up-regulated ribosome production down-regulates p53 expression, thus promoting neoplastic transformation [27]. Ribosome biogenesis is also involved in the relationship between chronic inflammation and cancer [28]. Epidemiological studies have shown that the reduced risk of cancer onset among regular non-steroidal anti-inflammatory aspirin drug users is very likely due to the perturbation of rRNA maturation [29, 30]. Our data suggest that up-regulated ribosome biogenesis is a representative function change in FC and likely occurs early in the spatiotemporal dynamics of carcinogenesis.

Since Warburg first suggested the metabolic switch in cancer cells, studies have highlighted the important role of reprogrammed metabolic pathways during the process of cellular transformation and cancer progression [31, 32]. Mitochondrial genomic mutation is an early marker of FC in head and neck squamous cancer, gastrointestinal cancer and prostate cancer [33–35]. Function analysis of the distance-related down-regulated key PPI clusters highlighted fatty acid metabolic processes, which reflect the function of the mitochondrion, one of the important changes in FC. The balance of fatty acid synthesis and oxidation is disrupted in cancer [36]. Highly proliferative cancer cells require increased fatty acid synthesis for building new membranes and producing steroid hormones to sustain cell growth [37]. Although fatty acid oxidation is much more effective in producing ATP than glycolysis, cancer cells prefer glycolysis to provide energy [31]. Studies of cancer metabolism have revealed that inhibition of the tumor suppressor p53 can activate fatty acid synthesis and inhibit fatty acid oxidation [38, 39]. Increasing evidence suggests that fatty acids play an immunomodulatory role in cancer progression [40, 41]. As shown in our study, fatty acid oxidation is inhibited in cancer tissues compared to normal tissues, and 57.14% (4/7) of the members of the cluster exhibited similar down-regulation trends from normal tissues, to adenomas and cancer tissues. Furthermore, 3 genes showed a relationship with the prognosis of patients with CRC in the TCGA dataset, and CPT2 was an independent factor predicting overall survival. CPT2 is the key enzyme in fatty acid oxidation and is located on the mitochondrial membrane. Studies have recently revealed that CPT1 can promote tumor growth in a T cell-dependent manner [40]. However, much less is known about the role of CPT2 in cancer. Our results show that cancer tissues down-regulate the expression of CPT2 and that higher expression of CPT2 in cancer tissue independently predicts better prognosis in CRC patients, indicating the important role of fatty acid oxidation, especially CPT2, in cancer progression.

Our study has several limitations. First, the samples used in our distance-related model were all from patients with rectal cancer, whereas the validation data included both colon cancer and rectal cancer samples. Second, although we observed the prognostic value of CPT2 in CRC patients, the molecular mechanism by which CPT2 affects cancer progression is unknown. Further investigations are needed. Third, we cannot arbitrarily consider the FC clusters identified in our study as representing the panorama of FC. We explored the functions of the key FC clusters using a GO enrichment analysis. An integration of the multi-omics data of TCGA datasets would contribute to further analyses of the potential regulatory pathways associated with FC [42]. Our data contained information about lncRNA expression that was not fully utilized. According to the study by Wang et al., small molecule drugs treat cancer by affecting miRNA expression, which is predicted based on functional similarities [43]. Therefore, studies exploring the relationship between FC-related lncRNA expression and the small molecule drugs might provide information for cancer therapy.

In conclusion, using a distance-related model constructed to study FC in rectal cancer patients, we identified key function changes in FC and found that up-regulated ribosome biogenesis and down-regulated fatty acid metabolism in cancer might occur early in carcinogenesis. Moreover, we identified CPT2 as an independent prognostic factor in CRC patients. Our findings suggest that deep investigation of FC in CRC may provide valuable information about oncogenesis and disease progression.

## MATERIALS AND METHODS

### Collection of clinical samples

The clinical samples were collected from 32 rectal cancer patients who underwent surgical resection at the National Cancer Center/Cancer Hospital, Chinese Academy of Medical Sciences, from 2014 to 2015. We collected rectal cancer tissue samples (T) and histologically normal tissue samples located at varying distances from the tumors, including 1 cm from the tumor (N1), 5 cm from the tumor (N5) and as far as possible from the tumor (proximal end of the resected sample, NP). Malignant and matched normal tissues from each patient were snap-frozen in liquid nitrogen and stored at -80°C for subsequent molecular analysis. The status of all tissue specimens was confirmed histologically. Clinicopathological information was obtained for all patients. The use of human samples for this study was approved by the Ethics Committee of the National Cancer Center/Cancer Hospital, Chinese Academy of Medical Sciences, under approval number CH-BMS-015.

### Microarray analysis

Total RNA was extracted using the RNeasy Mini kit (Qiagen, Germantown, MD, USA). The RNA was quantified using an ND-1000 UV-VIS Spectrophotometer (NanoDrop Technologies, Wilmington, DE, USA), and its integrity was assessed using an RNA 6000 LabChip kit in combination with an Agilent 2100 Bioanalyzer (Agilent, Santa Clara, CA, USA). RNA with integrity number (RIN) greater than 7.0 was used in this study. All samples were analyzed using an Agilent SurePrint G3 Human GE v2 8×60K Microarray (G4851B). All sample-labeling, hybridization and washing steps were conducted according to the manufacturer’s instructions. The slides were scanned with an Agilent SureScan Microarray Scanner, and the fluorescence intensities on raw images were read and processed to quantify data using Agilent Feature Extraction Software (v10.5.1.1). The raw data of gene features with median values greater than 100 were normalized by the median scale method using the R package “limma” [44]. An expression matrix with 14,913 gene features was used for the subsequent analysis.

We used R [45] and lme4 [46] to perform a linear mixed effects analysis of the relationship between gene expression and distance from the tumor. As a fixed effect, we entered the distance from the tumor into the model. As random effects, we entered intercepts for person for the effect of gene expression. *P*-values were obtained by likelihood ratio tests of the full model with the effect in question against the model without the effect in question. Genes with a false discovery rate (FDR) < 0.0001 were considered significantly distance-related differentially expressed genes that were gradually up-regulated or down-regulated based on the distance from the tumors.

Our laboratory previously constructed a multistep carcinogenesis model comprising gene expression profiles of 12 normal colon mucosa samples, 51 adenoma biopsy samples and 25 colon adenocarcinoma samples (GSE41657). We used a linear model to identify the gradually down-regulated or up-regulated genes from the normal colon mucosa, to adenoma and cancer tissues. A FDR < 0.05 was considered statistically significant. For the cross validation, the up-regulated and down-regulated genes in both models were intersected.

The raw and processed gene expression data and clinical information for the samples are publicly available in the Gene Expression Omnibus (GEO) database under series accession number GSE90627.

### PPI network construction and subnetwork analysis

STRING (Search Tool for the Retrieval of Interacting Genes/Proteins) was employed to construct PPI networks for distance-related up-regulated genes and down-regulated genes [47]. The networks were constructed using the default settings, which included only the high-confidence edges with STRING scores greater than 0.4. The tab-delimited format PPI networks were exported from STRING and imported into Cytoscape. We then used the “Molecular Complex Detection (MCODE)” Cytoscape plugin to identify discrete clusters (or modules/subnetworks) from the former PPI networks using default settings [48]. The top clusters (subnetworks) were screened under the conditions of minimum size=6 and minimum score=4. The subnetwork visualization and Gene Ontology (GO) functional enrichment analysis were conducted in STRING.

### Survival analysis

TCGA CRC RNA sequencing data used for the survival analysis were accessed using the R package “TCGA2STAT” [49]. We calculated 5-year overall survival for the 7-gene signature and for each single gene using data from the TCGA CRC dataset. Hierarchical clustering of the expression of the 7 genes divided the sample into two groups, which was used as a categorical variable to perform survival analysis. Kaplan-Meier survival analysis and the log-rank test were used to evaluate the prognostic value of the gene signature and single genes. The Cox proportional hazards regression model was used to evaluate the independence of the prognostic factors. The variables tested in the Cox regression analysis included age at time of diagnosis, gender, TNM stage and gene expression. A *p* value of < 0.05 was considered significant.

### Statistical analysis

All statistical tests were two-sided, and a 5% level of significance was used. The statistical analyses in this study were performed using R software (http://www.r-project.org).

## SUPPLEMENTARY MATERIALS FIGURES AND TABLES







## References

[1] Siegel RL, Miller KD, Jemal A (2017). Cancer statistics. CA Cancer J Clin.

[2] Markowitz SD, Bertagnolli MM (2009). Molecular origins of cancer: molecular basis of colorectal cancer. N Engl J Med.

[3] Slaughter DP, Southwick HW, Smejkal W (1953). Field cancerization in oral stratified squamous epithelium; clinical implications of multicentric origin. Cancer.

[4] Dakubo GD, Jakupciak JP, Birch-Machin MA, Parr RL (2007). Clinical implications and utility of field cancerization. Cancer Cell Int.

[5] Kadara H, Fujimoto J, Yoo SY, Maki Y, Gower AC, Kabbout M, Garcia MM, Chow CW, Chu Z, Mendoza G, Shen L, Kalhor N, Hong WK (2014). Transcriptomic architecture of the adjacent airway field cancerization in non-small cell lung cancer. J Natl Cancer Inst.

[6] Jakubek Y, Lang W, Vattathil S, Garcia M, Xu L, Huang L, Yoo SY, Shen L, Lu W, Chow CW, Weber Z, Davies G, Huang J (2016). Genomic landscape established by allelic imbalance in the cancerization field of a normal appearing airway. Cancer Res.

[7] Park SK, Song CS, Yang HJ, Jung YS, Choi KY, Koo DH, Kim KE, Jeong KU, Kim HO, Kim H, Chun HK, Park DI (2016). Field cancerization in sporadic colon cancer. Gut Liver.

[8] Cherkezyan L, Stypula-Cyrus Y, Subramanian H, White C, Dela Cruz M, Wali RK, Goldberg MJ, Bianchi LK, Roy HK, Backman V (2014). Nanoscale changes in chromatin organization represent the initial steps of tumorigenesis: a transmission electron microscopy study. BMC Cancer.

[9] Trujillo KA, Heaphy CM, Mai M, Vargas KM, Jones AC, Vo P, Butler KS, Joste NE, Bisoffi M, Griffith JK (2011). Markers of fibrosis and epithelial to mesenchymal transition demonstrate field cancerization in histologically normal tissue adjacent to breast tumors. Int J Cancer.

[10] Piccinin S, Gasparotto D, Vukosavljevic T, Barzan L, Sulfaro S, Maestro R, Boiocchi M (1998). Microsatellite instability in squamous cell carcinomas of the head and neck related to field cancerization phenomena. Br J Cancer.

[11] Gabriel KN, Jones AC, Nguyen JP, Antillon KS, Janos SN, Overton HN, Jenkins SM, Frisch EH, Trujillo KA, Bisoffi M (2016). Association and regulation of protein factors of field effect in prostate tissues. Int J Oncol.

[12] Jones AC, Antillon KS, Jenkins SM, Janos SN, Overton HN, Shoshan DS, Fischer EG, Trujillo KA, Bisoffi M (2015). Prostate Field cancerization: deregulated expression of macrophage inhibitory cytokine 1 (MIC-1) and platelet derived growth factor A (PDGF-A) in tumor adjacent tissue. PLoS One.

[13] Jothy S, Slesak B, Harłozińska A, Lapińska J, Adamiak J, Rabczyński J (1996). Field effect of human colon carcinoma on normal mucosa: relevance of carcinoembryonic antigen expression. Tumour Biol.

[14] Hawthorn L, Lan L, Mojica W (2014). Evidence for field effect cancerization in colorectal cancer. Genomics.

[15] Galandiuk S, Rodriguez-Justo M, Jeffery R, Nicholson AM, Cheng Y, Oukrif D, Elia G, Leedham SJ, McDonald SA, Wright NA, Graham TA (2012). Field cancerization in the intestinal epithelium of patients with Crohn's ileocolitis. Gastroenterology.

[16] Alonso S, Dai Y, Yamashita K, Horiuchi S, Dai T, Matsunaga A, Sánchez-Muñoz R, Bilbao-Sieyro C, Díaz-Chico JC, Chernov AV, Strongin AY, Perucho M (2015). Methylation of MGMT and ADAMTS14 in normal colon mucosa: biomarkers of a field defect for cancerization preferentially targeting elder African-Americans. Oncotarget.

[17] Shen L, Kondo Y, Rosner GL, Xiao L, Hernandez NS, Vilaythong J, Houlihan PS, Krouse RS, Prasad AR, Einspahr JG, Buckmeier J, Alberts DS, Hamilton SR (2005). MGMT promoter methylation and field defect in sporadic colorectal cancer. J Natl Cancer Inst.

[18] Svrcek M, Buhard O, Colas C, Coulet F, Dumont S, Massaoudi I, Lamri A, Hamelin R, Cosnes J, Oliveira C, Seruca R, Gaub MP, Legrain M (2010). Methylation tolerance due to an O6-methylguanine DNA methyltransferase (MGMT) field defect in the colonic mucosa: an initiating step in the development of mismatch repair-deficient colorectal cancers. Gut.

[19] Facista A, Nguyen H, Lewis C, Prasad AR, Ramsey L, Zaitlin B, Nfonsam V, Krouse RS, Bernstein H, Payne CM, Stern S, Oatman N, Banerjee B (2012). Deficient expression of DNA repair enzymes in early progression to sporadic colon cancer. Genome Integr.

[20] Lee KH, Lee JS, Nam JH, Choi C, Lee MC, Park CS, Juhng SW, Lee JH (2011). Promoter methylation status of hMLH1, hMSH2, and MGMT genes in colorectal cancer associated with adenoma–carcinoma sequence. Langenbecks Arch Surg.

[21] Polley AC, Mulholland F, Pin C, Williams EA, Bradburn DM, Mills SJ, Mathers JC, Johnson IT (2006). Proteomic analysis reveals field-wide changes in protein expression in the morphologically normal mucosa of patients with colorectal neoplasia. Cancer Res.

[22] Cisterna B, Biggiogera M (2010). Ribosome biogenesis: from structure to dynamics. Int Rev Cell Mol Biol.

[23] Derenzini M, Montanaro L, Trerè D (2017). Ribosome biogenesis and cancer. Acta Histochem.

[24] Thomas G (2000). An encore for ribosome biogenesis in the control of cell proliferation. Nat Cell Biol.

[25] Derenzini M, Montanaro L, Treré D (2009). What the nucleolus says to a tumour pathologist. Histopathology.

[26] Stefanovsky V, Langlois F, Gagnon-Kugler T, Rothblum LI, Moss T (2006). Growth factor signaling regulates elongation of RNA polymerase I transcription in mammals via UBF phosphorylation and r-chromatin remodeling. Mol Cell.

[27] Montanaro L, Treré D, Derenzini M (2012). Changes in ribosome biogenesis may induce cancer by down-regulating the cell tumor suppressor potential. Biochim Biophys Acta.

[28] Brighenti E, Calabrese C, Liguori G, Giannone FA, Trerè D, Montanaro L, Derenzini M (2014). Interleukin 6 downregulates p53 expression and activity by stimulating ribosome biogenesis: a new pathway connecting inflammation to cancer. Oncogene.

[29] Cao Y, Nishihara R, Wu K, Wang M, Ogino S, Willett WC, Spiegelman D, Fuchs CS, Giovannucci EL, Chan AT (2016). Population-wide impact of long-term use of aspirin and the risk for cancer. JAMA Oncol.

[30] Brighenti E, Giannone FA, Fornari F, Onofrillo C, Govoni M, Montanaro L, Treré D, Derenzini M (2016). Therapeutic dosages of aspirin counteract the IL-6 induced pro-tumorigenic effects by slowing down the ribosome biogenesis rate. Oncotarget.

[31] Koppenol WH, Bounds PL, Dang CV (2011). Otto Warburg's contributions to current concepts of cancer metabolism. Nat Rev Cancer.

[32] Carracedo A, Cantley LC, Pandolfi PP (2013). Cancer metabolism: fatty acid oxidation in the limelight. Nat Rev Cancer.

[33] Kim MM, Clinger JD, Masayesva BG, Ha PK, Zahurak ML, Westra WH, Califano JA (2004). Mitochondrial DNA quantity increases with histopathologic grade in premalignant and malignant head and neck lesions. Clin Cancer Res.

[34] Sui G, Zhou S, Wang J, Canto M, Lee EE, Eshleman JR, Montgomery EA, Sidransky D, Califano JA, Maitra A (2006). Mitochondrial DNA mutations in preneoplastic lesions of the gastrointestinal tract: a biomarker for the early detection of cancer. Mol Cancer.

[35] Parr RL, Dakubo GD, Crandall KA, Maki J, Reguly B, Aguirre A, Wittock R, Robinson K, Alexander JS, Birch-Machin MA, Abdel-Malak M, Froberg MK, Diamandis EP (2006). Somatic mitochondrial DNA mutations in prostate cancer and normal appearing adjacent glands in comparison to age-matched prostate samples without malignant histology. J Mol Diagn.

[36] Röhrig F, Schulze A (2016). The multifaceted roles of fatty acid synthesis in cancer. Nat Rev Cancer.

[37] Santos CR, Schulze A (2012). Lipid metabolism in cancer. FEBS J.

[38] Parrales A, Iwakuma T (2016). p53 as a regulator of lipid metabolism in cancer. Int J Mol Sci.

[39] Berkers CR, Maddocks ODK, Cheung EC, Mor I, Vousden KH (2013). Metabolic regulation by p53 family members. Cell Metab.

[40] Hossain F, Al-Khami AA, Wyczechowska D, Hernandez C, Zheng L, Reiss K, Valle LD, Trillo-Tinoco J, Maj T, Zou W, Rodriguez PC, Ochoa AC (2015). Inhibition of fatty acid oxidation modulates immunosuppressive functions of myeloid-derived suppressor cells and enhances cancer therapies. Cancer Immunol Res.

[41] Miccadei S, Masella R, Mileo AM, Gessani S (2016). ω3 polyunsaturated fatty acids as immunomodulators in colorectal cancer: new potential role in adjuvant therapies. Front Immunol.

[42] Jiang W, Jia P, Hutchinson KE, Johnson DB, Sosman JA, Zhao Z (2015). Clinically relevant genes and regulatory pathways associated with NRASQ61 mutations in melanoma through an integrative genomics approach. Oncotarget.

[43] Wang J, Meng F, Dai E, Yang F, Wang S, Chen X, Yang L, Wang Y, Jiang W (2016). Identification of associations between small molecule drugs and miRNAs based on functional similarity. Oncotarget.

[44] Ritchie ME, Phipson B, Wu D, Hu Y, Law CW, Shi W, Smyth GK (2015). Limma powers differential expression analyses for RNA-sequencing and microarray studies. Nucleic Acids Res.

[45] R Core Team (2016). R: a language and environment for statistical computing.

[46] Bates D, Maechler M, Ben Bolker S, Walker (2015). Fitting linear mixed-effects models using lme4. J Stat Softw.

[47] Szklarczyk D, Franceschini A, Wyder S, Forslund K, Heller D, Huerta-Cepas J, Simonovic M, Roth A, Santos A, Tsafou KP, Kuhn M, Bork P, Jensen LJ (2015). STRING v10: protein-protein interaction networks, integrated over the tree of life. Nucleic Acids Res.

[48] Bader GD, Hogue CW (2003). An automated method for finding molecular complexes in large protein interaction networks. BMC Bioinformatics.

[49] Wan YW, Allen GI, Anderson ML, Liu Z (2015). https://CRAN.R-project.org/package=TCGA2STAT.

