# Supplementary material for: Integrated transcriptomic analysis of distance-related field cancerization in rectal cancer patients
Source: Oncotarget. 2017 May 15;8(37):61107–17. doi: 10.18632/oncotarget.17864 (PMC5617410; doi:10.18632/oncotarget.17864)
Supplement: Supplementary file 1 [file oncotarget-08-61107-s001.pdf]

Integrated transcriptomic analysis of distance-related field cancerization in rectal cancer patients

SUPPLEMENTARY MATERIALS

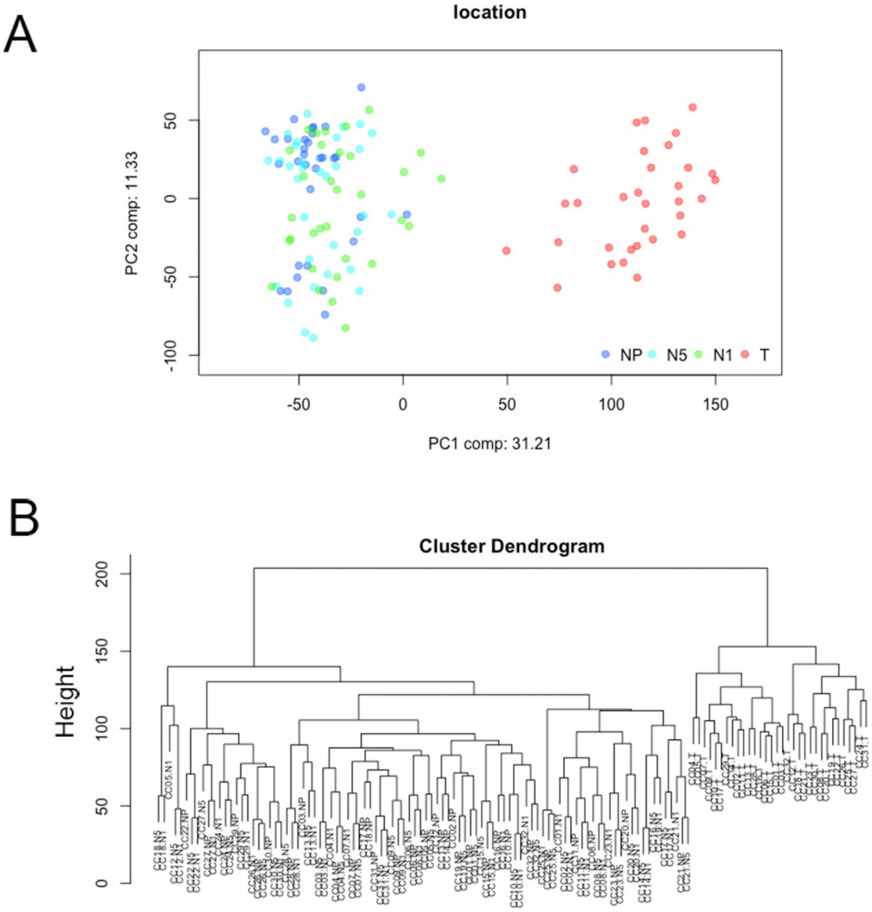

Supplementary Figure 1: PCA and hierarchical clustering of the data. X axis and Y axis of **(A)** represent the principle component 1 and 2 of the PCA, respectively. **(B)** shows the hierarchical clustering of the samples.

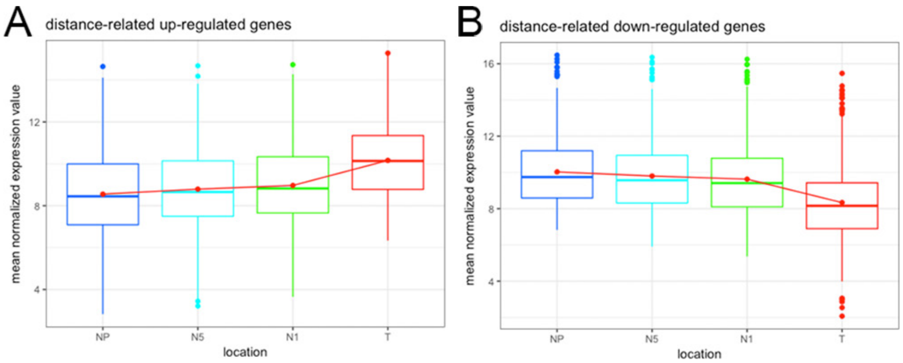

Supplementary Figure 2: Expression pattern of distance-related differentially expressed genes identified by linear mixed effects model. **(A)** and **(B)** show the gradually up regulated and down regulated genes, respectively.

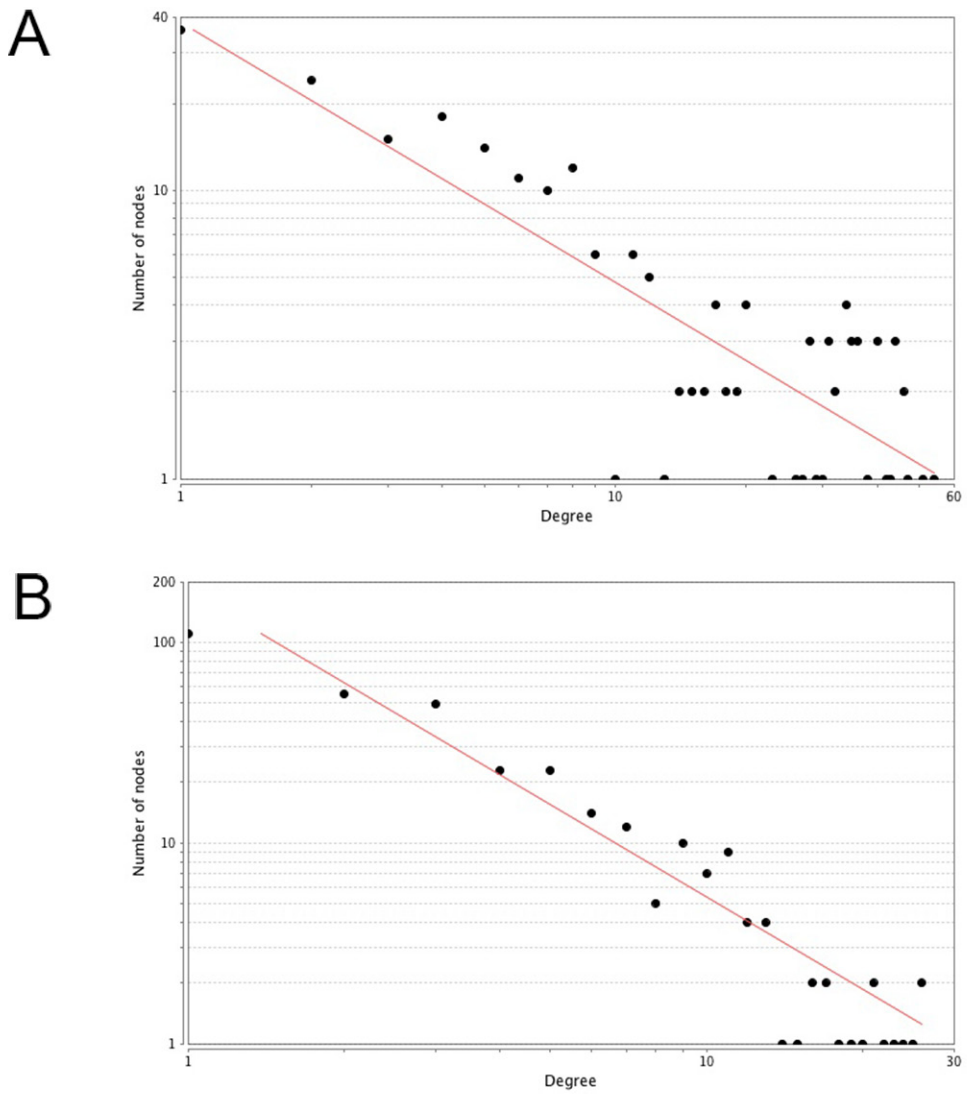

Supplementary Figure 3: Power law distribution of the nodes of the two PPI networks. (A) shows the node degree distribution of the up-regulated PPI network. **(B)** shows the node degree distribution of the down-regulated PPI network.

Supplementary Table 1: Clinical characteristics of the patients

No.	Age (year)	Gender	Diagnosis	NP (cm)	Polyps	Size (cm^2)	Differentiation	TNM
1	55	F	Rectal cancer	18	With	1.5	Well and mod	I
2	50	F	Rectal cancer	24	With	2	Mod	I
3	64	M	Rectal cancer	15	With	4.5	Mod	I
4	67	M	Rectal cancer	12	With	5.5	Well and mod	III
5	59	M	Rectal cancer	20	Without	4	Mod	I
6	48	M	Rectal cancer	7	With	3.8	Mod	III
7	47	M	Rectal cancer	8	With	5	Mod	II
8	60	F	Rectal cancer	25	Without	3.5	Mod	I
9	43	M	Rectal cancer	8	With	5.5	Mod	II
10	62	M	Rectal cancer	11	With	4	Mod	I
11	39	F	Rectal cancer	20	Without	2.5	Mod	III
12	68	F	Colorectal cancer	30	With	3.5	Mod	I
13	71	M	Rectal cancer	20	With	6.2	Well and mod	III
14	68	M	Rectal cancer	15	Without	9	Mod	II
15	50	M	Rectal cancer	15	With	4	Mod and poorly	I
16	79	M	Rectal cancer	6	Without	8	Mod	IV
17	47	M	Rectal cancer	10	Without	3	Mod	I
18	66	F	Rectal cancer	10	Without	4.5	Mod	II
19	76	M	Rectal cancer	8	Without	7.5	Mod	II
20	59	F	Rectal cancer	15	Without	4	Mod	II
21	43	M	Rectal cancer	15	Without	3	Mod and poorly	III
22	69	F	Rectal cancer	16	Without	5	Mod	III
23	46	F	Rectal cancer	6	With	3.8	Well	II
24	74	M	Rectal cancer	8	Without	3.5	Well and mod	III
25	56	M	Rectal cancer	12	With	4.5	Mod	II
26	74	F	Rectal cancer	10	Without	2.5	Mod	I
27	27	F	Rectal cancer	8	Without	6.4	Mod and poorly	I
28	63	M	Rectal cancer	6	With	3.5	Well and mod	III
29	63	F	Rectal cancer	15	Without	2.8	Mod	III
30	41	F	Rectal cancer	16	Without	5	Mod	II
31	70	M	Rectal cancer	7	Without	2	Mod	I
32	70	M	Rectal cancer	12	Without	5.8	Mod and poorly	III

Supplementary Table 2: Gene list of the distance-related up-regulated genes identified by linear mixed effects model analysis

See Supplementary File 1

Supplementary Table 3: Gene list of the distance-related down-regulated genes identified by linear mixed effects model analysis

See Supplementary File 2

Supplementary Table 4: Clinical characteristics of the multistep carcinogenesis samples

		Normal	Adenoma	Cancer
Gender	Male	7	34	18
	Female	5	17	7
	Total	12	51	25
Age (years)		46±14	60±15	65±12
Stage	I	−	−	9
	II	−	−	4
	III	−	−	11
	IV	−	−	1
